# Response of the tegument of *Fasciola hepatica* to infection and immunization sera *in vitro*

**DOI:** 10.1017/S003118202510022X

**Published:** 2025-06

**Authors:** Clive E. Bennett, Adam P.S. Bennett, Robert E.B. Hanna, Mark W. Robinson

**Affiliations:** 1 School of Biological Sciences, University of Southampton, Southampton, UK; 2 School of Biological Sciences, Queen’s University Belfast, Belfast, UK; 3 Department of Parasitology, Agrifood and Biosciences Institute, Belfast, UK

**Keywords:** extracellular vesicle, *Fasciola*, helminth, microvesicle, secretion, sera, tegument

## Abstract

The migratory phase is a critical time for *Fasciola hepatica* as it must locate, penetrate and migrate through the alimentary tract to the liver parenchyma whilst under attack from the host immune response. Here, scanning and transmission electron microscopy were used to monitor the *in vitro* effects of sera (with, and without, complement depletion) on *F. hepatica* newly excysted juveniles (NEJs) and flukes recovered at 7, 35, 70 and 98 days post infection (dpi) from the liver and bile ducts of rats. Test sera were from these *F. hepatica-*infected rats. A *F. hepatica* NEJ-specific rabbit antiserum was also used. All fluke stages demonstrated release of the tegumental glycocalyx and microvesicles and intense activity within the tegumental syncytium characterized by eccrine secretion of T-0/T-1/T-2 secretory bodies with subsequent microvillar formation and shedding of microvesicles from the apical plasma membrane. Exposure of both NEJs and 35 dpi flukes to 35 and 70 dpi rat sera produced significant amounts of eccrine-derived secretory material and putative attached immunocomplex. Rabbit anti-*F. hepatica* NEJ-specific antiserum produced similar responses at the NEJ tegument, including binding of putative immunocomplex to the surface, but with additional blistering of some regions of the apical plasma membrane. Our data suggest that immune sera stimulates multiple interrelated secretory mechanisms to maintain the integrity of the tegumental barrier in response to immune attack. Concurrent release of microvesicles may also serve to both divert the immune response away from the fluke itself and permit delivery of immunomodulatory cargo to immune effector cells.

## Graphical abstract

**Figure:**
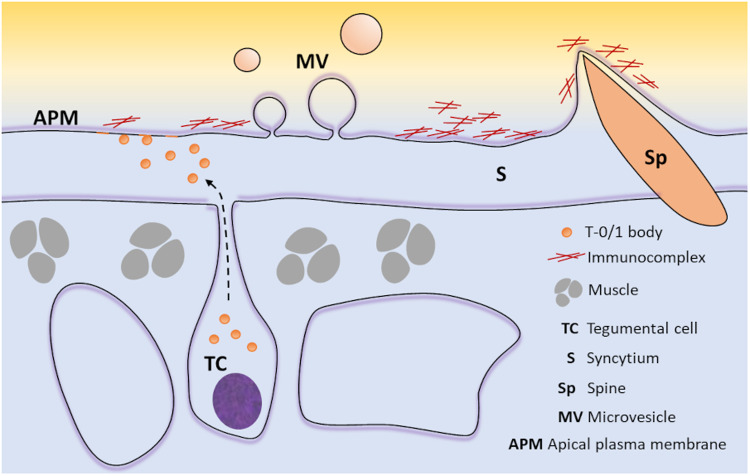

## Introduction

Stages of *Fasciola hepatica*, from early infection to patency (egg-laying), have a tegument comprised of a surface syncytium linked by cytoplasmic connections to 2 types of submuscularly located tegumental cell bodies which produce ellipsoid Type 1 (T-1) and flattened bi-concave Type 2 (T-2) secretory granules, respectively. Newly excysted juvenile (NEJ) flukes have a visibly different type of inclusion designated T-0, which appear more spherical (reviewed by Robinson et al., 2022). *Fasciola hepatica* recovered from the livers of rodents, and fixed for electron microscopy, have been observed to release tegumental apical plasma membrane by a form of eccrine secretion, whereby membrane and cytoplasm is also budded off (Bennett and Threadgold, 1975). This type of secretion has also been reported in hymenolepid tapeworms exposed to infection serum, and complement-depleted infection serum, *in vitro* (Hoole et al., 1994).

In this study, we investigate the phenomenon of tegumental eccrine secretion by *F. hepatica* in rats where migration to the bile duct takes approximately 70 days, and patency occurs soon afterwards. Strong resistance to reinfection (–98%) occurs in rats during the migration of a primary infection through the liver (Armour and Dargie, 1974; Hughes et al., 1981) with NEJs, transplanted intraperitoneally, being killed (Davies and Goose, 1981). Specifically, flukes at 28 days post infection (dpi) and adults both provoke resistance (Rajasekariah and Howell, 1978). Transfer of both immune, and hyperimmune, rat sera into naive animals, followed by challenge with metacercariae, provided evidence of protection against reinfection (Rajasekariah and Howell, 1979; Mitchell et al., 1981). Although heat treatment eliminated the protective effect of immune rat serum in passive transfer experiments (Hayes et al., 1974), complement component 3 does not bind to the surface of challenge flukes either *in vitro* or *in vivo* (Davies and Goose, 1981). We therefore sought to determine both quantitatively, and qualitatively, the effect of primary infection sera from rats, and juvenile antigen-specific sera from rabbit, on both *F. hepatica* NEJs and immature (liver stage) flukes *in vitro* with, and without, complement depletion.

## Materials and methods

### Parasites

*Fasciola hepatica* metacercariae were provided by the Veterinary Laboratories Agency (New Haw, UK) and either excysted *in vitro* to release NEJs, according to the method of Sewell and Purvis (1969), or used to orally infect female Wistar rats. Flukes were recovered from rat livers at 7, 35, 70 and 98 dpi and washed vigorously under a stream of NCTC-135 medium (Gibco, UK) for 30 s every 20 min followed by change into fresh medium over a period of 3 h to remove existing immunoprecipitate. Flukes were recovered from rat infections, since normal development of *F. hepatica* cannot be achieved to provide an equivalent stage *in vitro* without culture in serum (McCusker et al., 2016).

### Immune sera and incubation of F. hepatica in vitro

Serum was collected from the *F. hepatica*-infected rats at 7, 35, 70 and 90 dpi. Rabbit *F. hepatica* NEJ-specific antiserum, which had been adsorbed with whole adult fluke antigens, and shown to bind only to the tegumental surface of *F. hepatica* NEJs and early-stage immature flukes (Bennett et al., 1982) was also used. This adsorbed antiserum had been used to affinity-purify a set of juvenile-specific antigens which (with Freund’s Complete Adjuvant) resulted in a 63% reduction of adult fluke numbers at necropsy and a 66% reduction of liver damage by assessment of glutamate dehydrogenase levels (Bennett, 1986). Complement depletion, of both rat infection sera and rabbit *F. hepatica* NEJ-specific antiserum, was achieved by heat inactivation at 56°C for 30 min. As controls, we wished to determine the level of surface membrane release in medium alone and with normal (i.e. uninfected) rat and preimmune rabbit sera. Sera, with and without complement depletion, were added to 25 flukes at 30% (v/v) in NCTC culture medium (1 fluke/mL) for 1 h at 37°C without agitation. After incubation, the culture medium was removed and 20 flukes were processed for scanning electron microscopy (SEM) whilst 5 flukes were examined by transmission electron microscopy (TEM).

### Scanning electron microscopy

The amount of binding of putative immunoprecipitate to the surface of *F. hepatica*, following incubation with the various sera, was assessed using SEM according to Bennett (1975). Briefly, whole flukes were fixed by gentle addition of 6% glutaraldehyde in Millonig’s buffer for 1 h and dehydrated through an ethanol series and subjected to critical point drying with Freon 113. Samples were coated with gold and examined in a Cambridge Scanning Electron Microscope S4-10 operating at 20KV. The SEM prepared flukes were assessed for attached putative immunocomplexes by a subjective double-blind scoring of micrographs where – = 0% immunocomplex; + = 1–5% immunocomplex; ++ = 5–25% immunocomplex and +++ = >25% immunocomplex. These data were assessed by the Mann–Whitney *U* test for comparison between test and control sera.

### Transmission electron microscopy

Flukes were examined by TEM according to Bennett and Threadgold (1975). Briefly, flukes were fixed in 3% glutaraldehyde (w/v) in Millonig’s buffer (pH 7.2) containing CaCl_2_, and 3% sucrose (w/v), for 24 h and post-fixed in 1% aqueous osmium tetroxide for 2 h. Flukes were dehydrated in ethanol and embedded in Araldite epoxy resin (Ciba-Geigy, UK). Ultrathin sections were cut on a Reichert OMU2 ultramicrotome and double-stained in alcoholic uranyl acetate and lead citrate. Sections were examined with an AEI EM801 transmission electron microscope. Five flukes from each test and control treatment were sectioned from 2 areas: 1 anterior region and 1 region posterior to the ventral sucker. Typical surface features were recorded by 2 photomicrographs, taken at random points, in which each displayed 50% tegument and 50% space above the tegument (i.e. the area in which putative immunocomplex may be attached to the fluke outer surface). Photomicrographs (*n* = 20 for each serum tested) were assessed by (a) number of cases of secretion of eccrine-derived material in contact with a 5 µm length of the apical plasma membrane; (b) number of cases of released eccrine-derived material, apparently free of the apical plasma membrane (but within the immunocomplex), in a 20 µm^2^ area above a 5 µm length of the apical plasma membrane; (c) % of immunocomplex within a 20 µm^2^ area above a 5 µm length of the apical plasma membrane. Counting was conducted double blind and data compared by the Mann–Whitney *U* test.

## Results

### Control sera and medium

**Figure 1.**
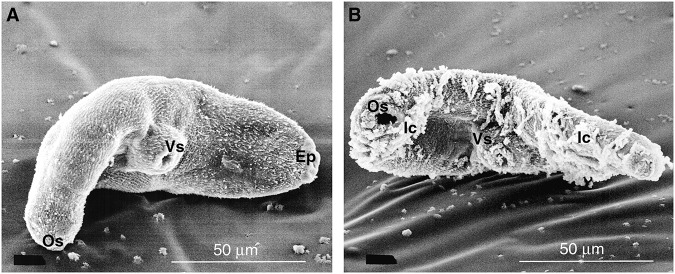
Scanning electron micrographs of *F. hepatica* NEJs after treatment with (A) uninfected control rat serum and (B) *F. hepatica*-infected (35 dpi) rat serum for 1 h *in vitro* at 37°C. Os, oral sucker; Vs, ventral sucker; Ep, excretory pore; Ic, putative immunocomplex.

**Figure 2.**
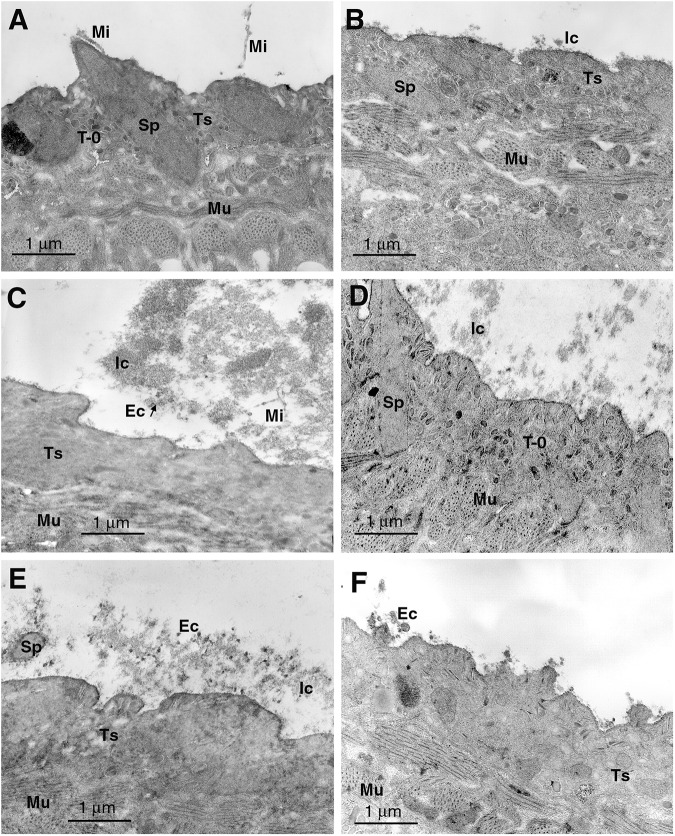
Transmission electron micrographs of *F. hepatica* NEJs exposed to (A) normal rat serum, (B) *F. hepatica*-infected 7 dpi rat serum, (C) heat-inactivated 7 dpi rat serum, (D and E) 35 dpi rat serum and (F) heat-inactivated 35 dpi rat serum for 1 h *in vitro* at 37°C. Ec, eccrine-derived material; Ic, putative immunocomplex; mi, microvillous eccrine secretion, Mu, muscle; Sp, spine; T-0, type 0 secretory granule; Ts, tegumental syncytium.

*Fasciola hepatica* NEJs and 35 dpi flukes incubated in medium alone, or with uninfected rat or preimmune rabbit sera, displayed normal tegumental morphology with only minimal surface reactions (Figs 1A, 2A and 4B). The attachment of putative immunocomplex to the tegumental surface of these flukes was negligible (Tables 1–4). No microvillar or lamellar membrane extensions of the apical plasma membrane were observed in NEJs fixed immediately post-excystment.

**Table 1.** Mean score of putative immunocomplex fixed in association with the tegument as viewed by SEM after exposure to medium alone or 30% serum in NCTC medium for 1 h (7 and 35 dpi rat serum compared with uninfected control rat serum)

	Treatment			
Flukes (dpi)	NCTC medium	Control rat serum	7 dpi rat serum	35 dpi rat serum
0 (NEJ)	+	+	++	++^a^
7	+	+	++	++^a^
35	+	+	++	+++^a^
70	+	+	+	++^a^
98	+	+	+	+

a Significantly different (*P* < 0.05) from control rat serum.

**Table 2.** Mean score (expressed as a percentage) of tegument response and putative immunocomplexes recorded by TEM after exposure to 30% serum in NCTC medium for 1 h (7, 35, 70 and 98 dpi rat sera compared with medium alone and uninfected control rat serum)

	Treatment					
Flukes (dpi)	NCTC medium	Control rat serum	7 dpi rat serum	35 dpi rat serum	70 dpi rat serum	98 dpi rat serum
0	2, 2, 4	1, 2, 2	0, 1, 25	2, 7, 31^a^	1, 38^a^, 27^a^	2, 1, 3
35	2, 4, 3	1, 6, 4	2, 15, 20	2, 30^a^, 29^a^	2, 30^a^, 29^a^	1, 1, 2

Data in order of presentation are (i) eccrine-derived material in contact with tegumental apical plasma membrane, (ii) eccrine-derived material found above the apical plasma membrane within the immunocomplex; (iii) % local immunocomplex in 20 µm^2^.

a Significantly different (*P* < 0.05) from control rat serum.

**Table 3.** Mean score of amount of putative immunocomplex fixed in association with the tegument after exposure to 30% serum in NCTC medium for 1 h for rabbit anti-*F. hepatica* NEJ-specific antiserum compared with preimmune rabbit serum

	Treatment		
Flukes (dpi)	NCTC medium	Rabbit preimmune serum	Rabbit anti-*F. hepatica* NEJ-specific serum
0 (NEJ)	+	+	+++^a^

a Significantly different (*P* < 0.05) from control rabbit serum.

**Table 4.** Mean score (expressed as a percentage) of tegument response and putative immunocomplex recorded by TEM after exposure to 30% serum in NCTC medium for 1 h (rabbit anti-*F. hepatica* NEJ-specific serum compared with medium alone and preimmune rabbit serum)

	Treatment		
Flukes (dpi)	NCTC medium	Rabbit preimmune serum	Rabbit anti-*F. hepatica* NEJ-specific serum
0 (NEJ)	3, 2, 3	5, 5, 4	5, 20^a^, 20^a^

Data in order of presentation are (i) eccrine-derived material in contact with the apical plasma membrane; (ii) eccrine-derived material found above the apical plasma membrane within the immunocomplex; (iii) % local immunocomplex in 20 µm^2^.

a Significantly different (*P* < 0.05) from control rabbit serum.

### SEM and TEM of flukes exposed to rat sera

Serum from *F. hepatica-*infected rats (7 and 35 dpi), both complement intact and depleted, produced visible surface responses when applied to NEJs and migratory juvenile (35 dpi) *F. hepatica* (Figure 1B, Table 1). When viewed by SEM, apparent immunocomplexes were seen to cover much of the surface of the flukes and were commonly seen attached to the tips of spines of NEJs (Figure 4A).

**Figure 3.**
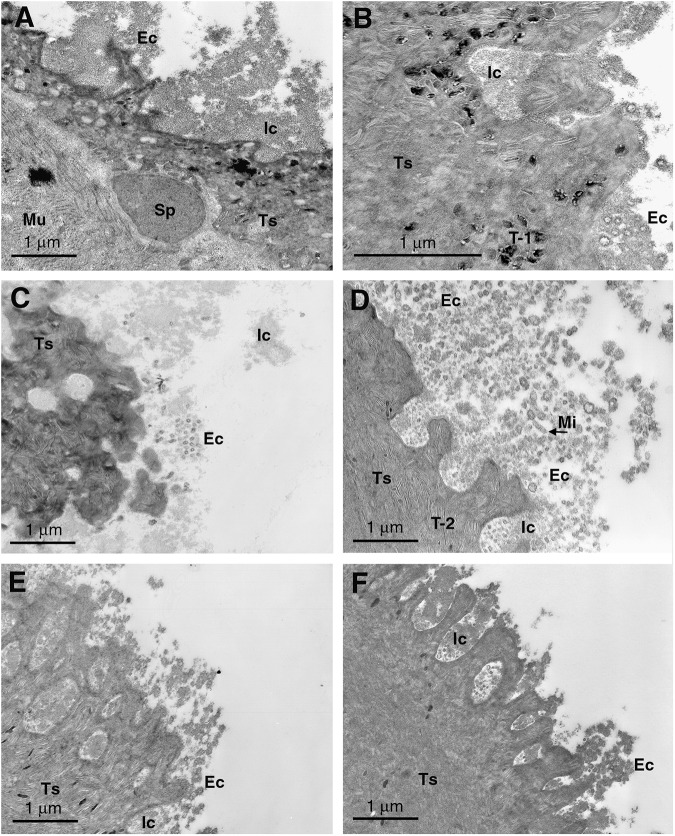
Transmission electron micrographs of juvenile *F. hepatica* recovered from rat liver and exposed to serum for 1 h *in vitro* at 37°C. (A) 7 dpi fluke exposed to *F. hepatica*-infected (35 dpi) rat serum, (B) 35 dpi fluke exposed to 35 dpi rat serum, (C) 35 dpi fluke exposed to heat-inactivated 35 dpi rat serum, (D) 35 dpi fluke exposed to 70 dpi rat serum, (E) 35 dpi fluke exposed to 98 dpi rat serum and (F) 35 dpi fluke exposed to heat-inactivated 98 dpi rat serum. Ec, eccrine-derived material; ic, putative immunocomplex; Mi, microvillar structures; Mu, muscle; Sp, spine; T-1, type 1 secretory granule; T-2, type 2 secretory granule; Ts, tegumental syncytium.

When viewed by TEM, it was evident that immune serum taken from infected rats (7 dpi and later), induced a response at the apical plasma membrane of the tegumental syncytium of *F. hepatica* NEJs. This was in the form of release of eccrine-derived material including T-0 granules and glycocalyx with apparent microvillar extensions and/or microvesicles in an extensive putative immunocomplex reaction (Figures 2C and 3D). The amount of response was significantly higher (*P* < 0.05) with rat infection sera than uninfected sera (Figure 2A). The largest and most dense concentration of microvillar extensions/microvesicles was found in reaction with 35 dpi flukes and the diameter of these structures increased with distance from the tegumental apical plasma membrane (Figure 3D).

**Figure 4.**
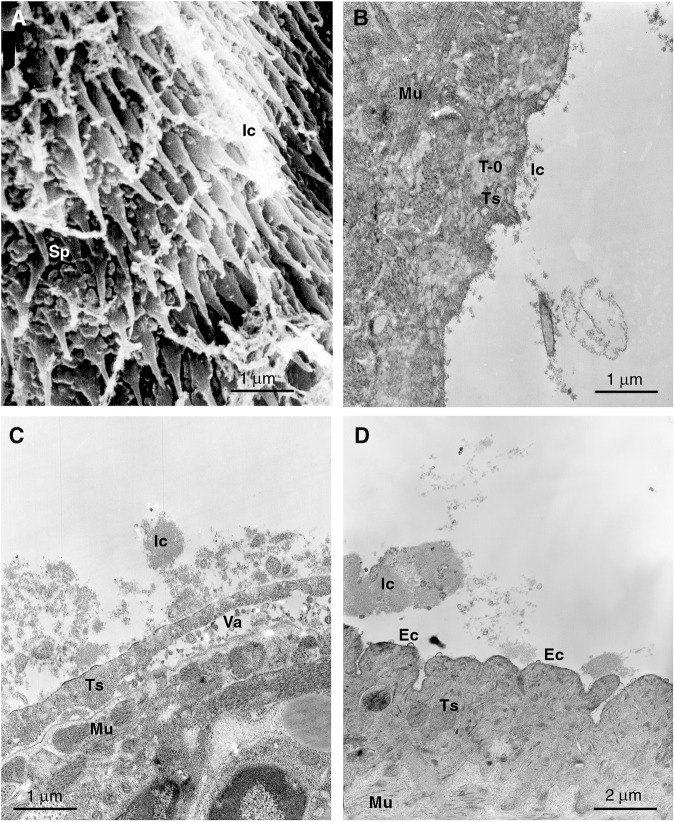
Electron micrographs of *F. hepatica* NEJs incubated with serum for 1 h *in vitro* at 37°C (A) scanning electron micrograph showing exposure to heat-inactivated *F. hepatica* NEJ-specific antisera. (B–D) transmission electron micrographs showing incubation with (B) normal rabbit serum, (C) *F. hepatica* NEJ-specific antiserum and (D) heat-inactivated *F. hepatica* NEJ-specific antiserum showing eccrine-derived microvesicle secretion (ec). Ic, putative immunocomplex; Mu, muscle; Sp, spine; Ts, tegumental syncytium; T-0, type 0 tegumental granule; Va, vacuolation.

### SEM and TEM of flukes exposed to NEJ-specific F. hepatica rabbit sera

When applied to the surface of infectious NEJs both complement intact and heat-depleted NEJ-specific immunization serum from rabbits produced evidence of surface responses when viewed by SEM (Figure 4A, Table 3) and by TEM (Figure 4B-D, Table 4). The nature of the eccrine secretions formed under the effect of immune serum was qualitatively different from that of rat infection serum, with the rabbit *F. hepatica* NEJ-specific antiserum-producing surface membrane release in the form of blebbing/microvesicle shedding from the apical membrane.

## Discussion

Extensive surface activity is reported here as a response to both immune and immunization serum on both *F. hepatica* NEJs and immature 35 dpi flukes. The data show that treatment with immune serum *in vitro* results not only in the formation of putative immunocomplexes on the tegumental surface, but also in the release of apical plasma membrane. This indicates that immune serum stimulates multiple interrelated secretory mechanisms to maintain the integrity of the tegumental barrier in response to immune attack. This results in the formation of what we here term ‘eccrine-derived material’ and propose a 2-step mechanism for its release at the apical plasma membrane. Firstly, eccrine secretion, referring to the release of non-membrane bound substances by exocytosis, occurs where secretory vesicles (in this case T-0/T-1/T-2 bodies) derived from tegumental cell bodies incorporate their membranes into the apical plasma membrane, and deposit their glycoprotein cargo at the glycocalyx (Garcia-Campos et al., 2016) with concurrent release of soluble secretory glycoproteins (De Marco Verissimo et al., 2023). This initial process occurs continually in *F. hepatica* to augment and replace the glycocalyx as immunocomplexes form and slough away (reviewed by Robinson et al., 2022). Secondly, microvesicle formation/blebbing occurs, where the apical plasma membrane protrudes outwards and eventually ‘pinches off’ to release membrane-bound vesicles containing apical cytoplasm into the extracellular (host) environment.

Blebbing of the tegument is thought to be a calcium-dependent process where membrane damage causes the influx of Ca^2+^ from the extracellular environment which drives Ca^2+^-dependent cytoskeletal re-modelling of the apical plasma membrane. This ultimately results in the release of microvesicles as a mechanism for tegument repair (de la Torre-escudero et al., 2016). The canonical molecular pathways for budding of extracellular vesicles (EVs) from the plasma membrane are conserved in *F. hepatica* and detected at the protein level in adult fluke EVs and the tegument (Bennett et al., 2020a, 2020b; Cwiklinski et al., 2015; de la Torre et al., 2019). Whilst the gut is thought to be the main source of *F. hepatica* EV secretion in adult flukes (Bennett et al., 2022; Bennett et al., 2020b), the extensive blebbing and release of material from the juvenile fluke tegument seen here in response to immune sera indicates that, *in vivo*, the juvenile tegument could be a major source of EV secretion in response to external host-derived stimuli encountered during migration. The beneficial release of EVs would explain the intense production of the T-0/T-1/T-2 bodies in the submuscular tegumental cells that contribute to the enormous amplification of the apical plasma membrane in response to putative membrano-glycocalyx sloughing in immunocomplexes with antibodies.

Eccrine secretions were at the highest levels with sera from rats recognized as resistant, 35 dpi, and with rabbit anti-NEJ *F. hepatica-*specific serum. The secretions were morphologically comparable to those described when flukes were transferred surgically to the peritoneal cavity of immune rats (Bennett et al., 1980). In these latter circumstances cell-mediated responses included the degranulation of eosinophils and had caused major tegumental disruption and decay, but the release of eccrine-derived material, as shown here, may have been a product of infection serum alone. From the results presented here, the turnover of the tegument under immune attack operates most extensively in 35 dpi flukes treated with 35 dpi serum (i.e. from the same animal) or with serum from older infections (70 dpi). The high level of eccrine secretion occurring during migration through the hepaticparenchyma is proposed to be a major immune avoidance mechanism (Hanna, 1980). Since *F. hepatica* EVs carry immunomodulators and have been shown to regulate host immune responses (Roig et al., 2018; Murphy et al., 2020) it would be of interest to determine the effects of the *in vitro* serum-induced microvesicles reported here, on host immune cell function. It is also feasible that these vesicles could act locally as a shield, or diversion, by removing tegumental antigens to a distance from the apical membrane. Conceivably, this could divert neutrophil function away from direct attack on the tegumental surface membrane. Of note, complement did not reduce the number of vesicular products of eccrine secretion during the experimental period (l h) and the significance of heat treatment in removing the protective effect of infection serum is not clear (Hayes et al., 1974). Nevertheless, our data provide further evidence of a defensive role of *F. hepatica* EVs and provoke a reassessment of previous interpretations of the release of the glycocalyx in the mid-developmental period (reviewed by Robinson et al., 2022) as a 2-step process involving initial eccrine secretion of T-0, T-1 and T-2 granules and subsequent microvesicle release from the apical plasma membrane.
